# Case of Puerperal Convulsions Attended with Rupture of the Uterus, and Recovery

**Published:** 1878-12

**Authors:** A. C. Rankin


### A CASE OF PUERPERAL CONVULSIONS, ATTENDED WITH RUPTURE OF THE UTERUS AND RECOVERY.

By A. C. Rankin, M. D.

(*Read before the Central Illinois Medical Association, in May*, 1878.)

On my return from the American Medical Association, at midnight, on June 9th, 1877, a messenger was waiting for me to visit Mrs. L., living eight miles in the country. Upon my arrival at the house, I found that Mrs. L. had been suffering from puerperal eclampsia since four o'clock of the previous evening. She was a large, powerful woman, aged twenty-three, and about seven months pregnant with her second child. She had had no trouble with her first confinement. Her spasms were very violent, and occurred at intervals of from fifteen minutes to one hour. She was unconscious from her first one, and violently delirious between her convulsions, so much so that it required four men to hold her or to keep any clothing upon her. She was also suffering from general anasarca, which proved that her convulsions were caused by uræmic poison.

As it was impossible to get her to swallow medicine, I bled her very freely, but it made no impression whatever upon her convulsion or delirium. I pointed my finger nail and inserted it through the os and ruptured the membranes, in order to induce premature labor. I then went home for chloroform and obstetric case. Upon my return labor had commenced, and I assisted by dilating the os with my fingers. The dilation was slow and tedious. She had no convulsions while under the influence of chloroform, and her pains occurred at about every ten or fifteen minutes. After a few hours the chloroform gave out, and her spasms and delirium returned as violent as before.

June 10th. At 4 p.m. the os was dilated so that I could have performed craniotomy, but it was impossible to hold her hips still enough to render the operation safe. About one hour later, while I was oiling my arm, intending to turn and deliver, she took a violent spasm and pain both at the same time; suddenly both ceased, and she settled back in the bed in a collapsed condition. Supposing that she was dying, I applied ammonia to her nostrils, and poured whiskey into her mouth. As soon as respiration was established, I introduced my hand and found the child had passed through a rent on the left side of the uterus into the cavity of the abdomen to its armpits. Seizing the feet, I soon brought it to the world stillborn. The patient was meanwhile lying in a semi-comatose condition. Upon reaching for the secundines, I found them also in the cavity of the abdomen. After removing them, I introduced my hand, leaving my thumb in Douglas' cul-de-sac, and my fingers in the abodomen, and scraped out a quantity of blood and clots, cleaning the cavity of the abdomen as well as possible, and then replaced about a foot of the small intestines, which had escaped into the uterine cavity.

By that time the uterus had begun to contract. I kept the back of my hand against the rent, and held back the intestines. In about half an hour the uterus contracted so as to expel my hand. The rupture commenced about 2½ centimetres above the utero-vaginal connection, and extended up the left side about eight inches. At the time I removed my hand the rent was between 6 and 8 centimetres long.

June 11th. Returned early in the morning; found patient quiet but still unconscious, having had no convulsions since she was delivered of the child; pulse 67. She was still suffering from the shock; would eagerly swallow anything placed in her mouth, and appeared hungry. Ordered beef tea, and a little hot water and whiskey. Remained with her all day; by evening she had rallied somewhat; pulse 80.

June 12th. Patient had become conscious during the night; pulse 90; complained of pain in left inguinal region; ordered a turpentine stupe, and gave morphine with quinine.

June 13th. A large dose of oil was given to her in the night without my knowledge; however, it operated without doing any apparent harm; pulse 105; pain in her side about the same; a cake as large as my hand had formed at the seat of the pain. Ordered acetate of potassa, and continued the morphine and turpentine stupes.

June 14th. Was called in the night; arrived at 3 a.m.; pulse 135; abdomen very much enlarged and tympanitic; she was suffering from general peritonitis. I made a solution of tincture of veratrum viride with water, and gave her a teaspoonful every hour. In four hours it reduced her pulse to less than 100. I then gave three centigrams of morphine every two hours. The second dose relieved her pain, after which the dose was reduced and given every two to three hours, alternating with the veratrum. I had her sponged off twice each day, her bed kept perfectly clean, and her napkins saturated with a solution of carbolic acid. I remained with her all day, watching the effects of the veratrum, and keeping her pulse at from 95 to 100. Her stomach remained in good condition, retaining medicine and nourishment.

June 15th. Patient worse; bowels very tympanitic and tender; pulse 110; stopped morphine and gave rhubarb and bicarbonate of soda. It operated by noon, greatly relieving the tympanitis. Soda and morphine were next given, and turpentine stupes continued.

June 16. Pulse 95; bowels reduced but still tympanitic. Cake still in left inguinal region.

June 18th. Patient had a chill but no fever, and slight rigors during the day. Cake in her side soft and fluctuating.

June 19th. Had a cold, clammy sweat; gave whisky and quinine.

June 20th. An abscess broke, and about four ounces of thick pus were discharged per vaginam. Cake in her side had disappeared.

June 21st. Patient evidently convalescent. From this time she improved and made a good recovery. Her dropsy had gradually disappeared.

In December, 1877, she aborted at about the third month. No physician was called, and I have not learned the particulars. I have seen her since, however, and she appears to be in good health.

P. S. Since the above was read, I was called Oct. 31, 1878, to see Mrs. L. I found her in labor; in a few hours she was delivered of a healthy child, weighing seven pounds. Her labor was natural, pains regular and quite severe, but two of them being required to expel the child after its head had passed the uterus.

---

THE HORRORS OF TEETH FILLING, with the preliminary gouging and filing, are to many so unpleasant that they prefer the pain of extraction to the annoyance of filling. In future, they may take their choice without the necessity of a sacrifice of the tooth, if they prefer extraction. Dr. Weil, of Munich, has employed and advocated the method of first extracting the tooth, filling it with gold or amalgam, and then replacing it. He states that the results are excellent, and the teeth can be freely used. He keeps the tooth out of its socket for one or two hours, as may be necessary, and yet the tooth is ultimately firmly fixed. The method is applicable to both bicuspids and molars, and will probably be chosen by many persons, provided it is found as successful in other hands as in those of the inventor.

